# Definitions of “Mental Disorder” from DSM-III to DSM-5

**DOI:** 10.3390/bs15060830

**Published:** 2025-06-18

**Authors:** Mattia Vacca, Alessandro Mura, Gian Pietro Carrogu, Luca Gaviano, Riccardo Atzori, Donatella R. Petretto

**Affiliations:** Department of Education, Philosophy and Psychology, State University of Cagliari, 09124 Cagliari, Italy; mattiavacca67@gmail.com (M.V.); alessandro.mura@pulimpiu.com (A.M.); gpcarrogu@gmail.com (G.P.C.); luca.gvn@gmail.com (L.G.); riccardoatzori@hotmail.it (R.A.)

**Keywords:** mental disorder, DSM, definition, psychopathology

## Abstract

The definition of “mental disorder” (MD) is one of the most critical themes throughout the history of psychopathology and in the development of the discipline itself. Despite this theme having been addressed since ancient times, the first explicit and shared definition of MD only appeared in the seventies, soon after the first internationally shared nosographies. In this perspective paper, we focused on the definitions of MD proposed in the various versions of the “Diagnostic and Statistical Manual of Mental Disorders—DSM”, starting from the third edition of 1980. Over about 40 years, in the various versions of the DSM, six definitions of MD were proposed. We discussed the general matrix/structure of those definitions, as well as the main similarities and/or divergences, and some of the specific constructs and approaches used in such definitions. Additionally, we examined some papers that addressed the same topic in a similar manner and discussed the general debate that accompanied the proposal of the various DSM definitions, the significant attention those definitions attracted, and some minor and major criticisms.

## 1. Introduction

The definition of “mental disorder” (MD) is undoubtedly one of the most critical themes throughout the history of psychopathology and in the development of the discipline itself. Despite this theme having been addressed since ancient times, clinicians and scholars have only recently become aware of the need to share common languages and nosology, and it was not until recent decades that they also became more aware of the need to achieve a shared definition of the construct “mental disorder”. For these reasons, the first explicit and shared definition of “mental disorder” only appeared in the seventies, and soon after, the first internationally shared nosographies were also elaborated, thanks to the efforts of the American Psychiatric Association and the World Health Organization (with the development of the Diagnostic and Statistical Manual of Mental Disorders—DSM, and International Classification of Diseases—ICD). However, regarding international shared nosographies, the first definition of “mental disorder” appeared in the third version of the Diagnostic and Statistical Manual (DSM) by the American Psychiatric Association (APA, 1980). It was the first manual to explicitly provide a definition of MD (Petretto et al., 2025), following the publication of Spitzer and Endicott’s definition (Spitzer & Endicott, 1978). That is when a complex and still open debate on the definition of mental disorder started, soon after the publication of Spitzer and Endicott’s definition (Spitzer & Endicott, 1978); it proceeded in the following years. In defining the concept of mental disorder, Spitzer and Endicott (1978) addressed the concept of medical disorder first, describing it as “*a relatively distinct condition resulting from an organismic dysfunction which in its fully developed or extreme form is directly and intrinsically associated with distress, disability, or certain other types of disadvantage. The disadvantage may be of a physical, perceptual, sexual, or interpersonal nature. Implicitly there is a call for action on the part of the person who has the condition, the medical or its allied professions, and society*”, then, the authors proceeded to discuss mental disorder as a subset of medical disorder: “*A mental disorder is a medical disorder whose manifestations are primarily signs or symptoms of a psychological (behavioral) nature, or if physical, can be understood only using psychological concepts*.” The inclusion of such a definition in the first draft of the DSM-III (APA, 1980) caused a series of controversies between the American Psychiatric Association and the American Psychological Association, which was concerned about the jurisdiction problems raised by considering mental disorders a subset of medical disorders. The matter of the controversy can be summarized as follows: if mental disorders are medical disorders (as argued by Spitzer & Endicott, 1978), should they be treated by physicians and/or psychiatrists only? Such controversies eventually led the American Psychiatric Association to abandon any reference to mental disorders being medical disorders in the final draft of the DSM-III (APA, 1980). The DSM-III’s (APA, 1980) definition of mental disorder attracted the interest of many authors, such as in the case of Wakefield’s two seminal papers of 1992 (Wakefield, 1992a, 1992b) that opened up a complex and still ongoing debate about the definition of mental disorder (Petretto et al., 2025). In this brief perspective paper, we focused on the definitions proposed in the Diagnostic and Statistical Manual (DSM), starting from DSM-III’s first provided definition of mental disorder (APA, 1980) to all the others proposed in the subsequent DSM editions (DSM-III-R, DSM-IV, DSM-IV-TR, DSM-5, and DSM-5-TR) (APA, 1987, 1994, 2000, 2013, 2022). In the analysis, we focused on both the points of contact and divergences between the proposed definitions. More in detail, the purpose of this paper is to highlight if the DSM’s mental disorder definitions have changed in the transitions between the various editions and how these changes (if any) are, as well as to show the main issues of analysis that have been taken into consideration by other authors involved in the debate about the definition of mental disorder. Table 1 shows the DSM’s proposed definitions (italics shows specific changes in each definition).

## 2. Similarities Between the Six Definitions of Mental Disorder Proposed in the DSM

As shown in Table 1 and Table 2, all the DSM’s proposed definitions share a similar structure: they refer to an overall description of MD’s features, they describe a series of consequences of MD in individual life, and they all refer to underlying mechanisms. With regard to the cited specific features, mental disorder is proposed to be “*a clinically significant behavioral or psychological syndrome or pattern that occurs in an individual*” in DSM-III, DSM-III-R, DSM-IV, and DSM-IV-TR (APA, 1980, 1987, 1994, 2000); while, in DSM-5 and DSM-5-TR, mental disorder is described as “*a clinically significant disturbance in an individual’s cognition, emotion regulation, or behavior*” (APA, 2013, 2022). On this matter, it is noteworthy that while in the first proposed definitions in DSM-III and DSM-IV (and in their revised versions as well) the terms “psychological” and “behavioral” are chosen to describe MD’s general features (APA, 1980, 1987, 1994, 2000), in the following definitions, a more specific description of the involved psychological domains is proposed (cognition, emotion regulation, and behavior) (APA, 2013, 2022). This shift from general features to psychological domains is also based on a theoretical shift and on the change in the focus on “dysfunction” as the primary feature of mental disorder.

Regarding the consequences on individuals’ lives, in DSM-III and DSM-III-R (APA, 1980, 1987), the authors referred to “*distress or disability in one or more important areas of functioning*”, and specified that such consequences are “*typically associated*” in DSM-III (APA, 1980) and “*associated*” in DSM-III-TR (APA, 1987) with MD. Moreover, since DSM-III-R and until DSM-IV-TR, the aforementioned consequences were also associated with “*a significantly increased risk of suffering death, pain, disability, or an important loss of freedom*” (APA, 1987, 1994, 2000). Moreover, in DSM-5, mental disorder is once again described as “*usually associated*” with “*significant distress or disability in social, occupational, or other important activities*” (APA, 2013). In this respect, while the cited negative consequences are “*typically associated*” with MD in the first DSM-III definition (APA, 1980), and “*associated*” with MD in DSM-III-R, DSM-IV, and DSM-IV-TR (APA, 1994, 2000), in the most recent DSM-5 and DSM-5-TR definitions, such consequences are described as “*usually associated*” (APA, 2013, 2022), seemingly reintroducing a sort of cautionary approach about the relationship between MD and the described consequences. Such a choice could have been influenced by the intense debate on the consequences of MD and the relationship between MD and concepts like “harm”, “distress”, and “disability”, or “impairment” and “functioning” (which is better addressed in Petretto et al. (2025)).

With regard to the underlying mechanisms of MD, in DSM-III, the authors declared that “*there is an inference that there is a behavioral, psychological, or biological dysfunction*” (APA, 1980), while in DSM-III-TR, such a requirement was reformulated as “*whatever its original cause, it must currently be considered a manifestation of a behavioral, psychological, or biological dysfunction in the person*” (APA, 1987), and then this statement was identically maintained in DSM-IV and DSM-IV-TR (APA, 1994, 2000). As for the shift from general features to psychological domains, this other shift is based on a theoretical choice, where dysfunction is considered the primary feature of mental disorder. The following DSM editions, like the DSM-5 and DSM-5-TR, preferred the following formulation instead: “*(MD) reflects a dysfunction in the psychological, biological, or developmental processes underlying mental functioning*” (APA, 2013, 2022). In the first DSM-III definition, the authors used a very cautionary approach in referring to an underlying dysfunction (note that they used the expression “*there is an inference*”) (APA, 1980), and described three possible kinds of dysfunction (behavioral, biological, and psychological); in the following definitions, the authors referred in a similarly vague way to the “*original causes*” of MD, and affirmed that mental disorder “*must currently be considered a manifestation of a behavioral, psychological, or biological dysfunction in the person*”, adopting a much less cautionary approach (APA, 1987, 1994, 2000). Then, in the DSM-5 and DSM-5-TR definitions (APA, 2013, 2022), the authors chose a different formulation and declared that the previously described “*clinically significant disturbance*” in the three cited specific domains (cognition, emotion regulation, or behavior) “*reflects a dysfunction in the psychological, biological, or developmental processes underlying mental functioning*” (note again the way in which the authors mentioned three supposedly diverse kinds of processes “*underlying mental functioning*”).

All the proposed definitions share some other common features useful for distinguishing mental disorder from other kinds of human facts/behaviors and conditions, such as the relationship between the individual and society vs. MD; social deviance vs. MD; and “*expectable and culturally sanctioned response to a particular* event” vs. MD. All the proposed definitions specify that MD is a “disturbance” or a “dysfunction” in the individual (or in the person), and that such disturbances or dysfunctions must be distinguished from conflict between the individual and society and socially deviant behavior (whether “*political, religious, or sexual*”). All the proposed DSM definitions, since DSM-III-R (APA, 1987), highlight that an “*expectable and/or culturally sanctioned response*” *to* “*a particular event*” (APA, 1987, 1994, 2000) or to “*a common stressor or loss*” (APA, 2013, 2022), is different from an MD.

## 3. Differences Between the Six Definitions of Mental Disorder Proposed in the DSM

Together with the cited main similarities between the various DSM proposed definitions, some divergences are also identifiable: first, terminologies and semantics used in each definition changed, both with reference to the levels of psychological functioning and domains, and with reference to the mechanisms/processes underlying mental disorders (the proposed definitions range from explicit reference to some specific causes and mechanisms/processes of mental disorder, to vague reference to presumably multilevel causal models); and with reference to the overall and specific consequences of MD on individual’s life (ranging from disability, functional impairment and pain or distress, to “*increased risk of … death, pain, disability … loss of freedom*”). Moreover, during the course of the definition development in the various DSM editions, some words appeared, then disappeared and later made a comeback, and few words seem to be used in such an interchangeable manner (like in the cases of the words “pain” and “distress” or “impairment” and “disability”). In this regard, it is even more noticeable that some specific words used in the cited definitions would require a proper definition themselves and may merit a deeper focus on their meaning (such in the case of the words “disability”, “functioning”, “distress”, and “developmental processes”). A great debate is still open regarding those specific words (see, for example, the still open debate on the construct “disability”). Sometimes, and risking to create even more confusion, the same idea seems to be expressed with different adverbs (e.g., “usually” or “typically”), maybe with the aim of highlighting different degrees of relation between the features of mental disorder and its causes/processes and/or consequences, or maybe aiming to propose different degrees in the frequency of specific features/characteristics (see Table 1 and Table 2).

## 4. Debate on the Six Definitions of MD Proposed in the DSM

The six DSMs’ definitions proposed from 1980 to 2022 have attracted much attention and activated a rich debate to which several authors have contributed (see, for example, Frances et al., 1990; Stein, 1991; Wakefield, 1992a, 1992b, 2003; Lilienfeld & Marino, 1995; Follette & Houts, 1996; Phillips, 2000; Crowe, 2000; Houts, 2001a, 2001b; Bolton, 2001; Jablensky, 2005; Berganza et al., 2005; Bolton, 2007; First & Frances, 2008; Stein et al., 2010; First & Wakefield, 2010; Malón, 2012; Horwitz, 2017).

In more detail in the following lines, we will discuss in a deeper way some of the cited points, using a chronological approach. In 1990, Frances et al. (1990) provided a historical overview of the development of international diagnostic manuals and discussed the in-progress work on the DSM-IV (APA, 1994). They highlighted the problematic conceptual divide between mental and physical disorders, promoted by the presence of a separate axis for physical disorders (axis III) in the DSM-III-R (APA, 1987), and the various controversies surrounding using terms such as “psychiatric disorders” or “mental disorders”, as in all DSM editions from the DSM-I (APA, 1952) to the DSM-5-TR (APA, 2022). Furthermore, Frances et al. proposed a future shift in the development of the DSM-IV (APA, 1994) toward a more prototypical view of mental disorders (Frances et al., 1990). They argued that if diagnostic categories are seen as prototypes of certain mental disorders rather than as heterogeneous categories with the same features, mental health professionals will be less likely to reify disorders and more likely to identify boundary cases with varying degrees of similarity to a defined prototype (Frances et al., 1990). Lastly, the authors (Frances et al., 1990) addressed the problem of polythetic versus monothetic criteria and pointed out the increased use of polythetic criteria in the DSM-III-R (APA, 1987). The following year, Stein (1991) specifically discussed the DSM-III’s (APA, 1980) definition of mental disorder and argued that such a definition takes into place a positivistic view of disorder, according to which both physical and mental diseases require a dysfunction, and the notion of disease is value-neutral. Then, Stein stated that even if the categorization of mental disorders necessarily entails some theory of the structures and mechanisms that generate the phenomenon (e.g., dysfunction), such categorization occurs within a sociohistorical context that must be taken into account (Stein, 1991). Furthermore, he mentioned the DSM-IV (APA, 1994), which was not published yet at the time, hoping for an improvement on previous classifications, and highlighted the problematic nature of classifying disorders as “organic” or “functional”, a proposal which was being discussed by the DSM-IV (APA, 1994) task force (Stein, 1991).

In 1992, Jerome Wakefield published the two just cited seminal papers (Wakefield, 1992a, 1992b), in which he specifically referred to the DSM-III-R’s (APA, 1987) definition of mental disorder (based on the work of Spitzer & Endicott, 1978) and discussed its conceptual validity. The author highlighted eight main points of interest in the said definition: (1) the idea of “clinical significant conditions” as a base for mental disorder, (2) the phrase “behavioral or psychological syndrome or pattern”, (3) the phrase “in the person”, (4) the phrase “is associated with”, (5) the list of potential disorder-related negative consequences indicated by the definition, (6) the phrase “it must currently be considered”, (7) the reference to biological dysfunction, and (8) the requirement that deviance from or conflict with society is not in itself a disorder (Wakefield, 1992a). Then he stated that the DSM-III-R’s (APA, 1987) definition of mental disorder contains two fundamental principles: that a disorder is a condition that has negative consequences for the person and that a disorder is a dysfunction. That said, according to Wakefield, by failing to indicate what dysfunction effectively is, the DSM-III-R’s (APA, 1987) definition fails to validly distinguish disorders from non-disorders (Wakefield, 1992a). Thus, he proposed various changes to the discussed DSM-III-R’s (APA, 1987) definition, formulating the so-called “harmful dysfunction analysis” of mental disorder. In the second paper, Wakefield (1992b) described his proposed definition as an “*hybrid account of disorder as harmful dysfunction, wherein dysfunction is a scientific and factual term based in evolutionary biology that refers to the failure of an internal mechanism to perform a natural function for which it was designed, and harmful is a value term referring to the consequences that occur to the person because of the dysfunction and are deemed negative by sociocultural standards.*” (p. 374). Wakefield also discussed six different points of view that highlight various problems with the concept of mental disorders, including whether the concept of mental disorder exists itself, disorder as a pure value concept, disorder as whatever professionals treat, disorder as statistical deviance, disorder as a biological disadvantage, and disorders as unexpectable distress or disability. Lastly, the author argued that his “harmful dysfunction analysis” might avoid the cited problems (Wakefield, 1992a, 1992b).

In 1995, Lilienfeld and Marino mentioned DSM-II (APA, 1968), DSM-III (APA, 1980), DSM-III-R (APA, 1987), and DSM-IV (APA, 1994), and argued that the next editions of the DSM (or other similar diagnostic manuals) should refrain from classifying psychopathological conditions as either disorders or non-disorders. The authors proposed instead that international diagnostic manuals should have the more pragmatic goal of providing a compilation of “*well-validated conditions that are currently deemed to require intervention (i.e., treatment, prevention, or both) by mental health professionals*” (Lilienfeld & Marino, 1995, p. 418).

In the following year, Follette and Houts (1996) discussed Spitzer and Endicott’s (1978) proposed mental disorder definition for the DSM-III (APA, 1980), in which mental disorder was described as “*a medical disorder whose manifestations are primarily signs or symptoms of a psychological (behavioral) nature, or if physical, can be understood only using psychological concepts*” (Spitzer & Endicott, 1978, p. 18). Follette and Houts highlighted the way Spitzer and Endicott’s proposal to include such a definition in the third edition of the DSM had the result of alimenting the professional jurisdiction problem between psychiatrists and psychologists. In fact, if mental disorders are medical disorders (as argued by Spitzer & Endicott, 1978) should they be treated by physicians and psychiatrists only? This proposal caused a series of controversies between the American Psychiatric Association and the American Psychological Association, and eventually led the American Psychiatric Association to abandon any reference to mental disorders being medical disorders in the final draft of the DSM-III (APA, 1980) (Follette & Houts, 1996). Follette and Houts also pointed out how the authors of the DSM-IV (APA, 1994) almost kept the same definition used in the DSM-III (APA, 1980) and DSM-III-R (APA, 1987), arguing the need to “*guide decisions regarding which conditions on the boundary between normality and pathology should be included in DSM-IV*” (APA, 1994, p. xxi). Then, even if the authors accused the DSM of maintaining a hidden medical model while claiming to be atheoretical, they acknowledged the importance of defining mental disorder. As stated by the authors, “*one cannot begin to develop a taxonomic system without an inclusive definition of what the object of study is*” (Follette & Houts, 1996, p. 1122). Nonetheless, Follette and Houts criticized both the DSM’s definition and Wakefield’s (1992a, 1992b) “harmful dysfunction analysis”, which was proposed to enforce the conceptual validity of that definition. The authors then stated that the proliferation of categories in recent editions of the DSM (such as the DSM-IV (APA, 1994)) is an indication that the classification system is inadequate and unlikely to produce scientific progress, and argued that different theoretically driven taxonomies should be allowed to freely compete based on how successful they are at achieving goals such as understanding the etiology, course, and the treatment of conditions (Follette & Houts, 1996).

In 1997, Bergner, discussing the DSM-IV’s (APA, 1994) definition of mental disorder, argued that such a definition is defective in several ways. According to the author, the DSM-IV’s (APA, 1994) definition essentially states that “*X is some Y whose characteristics we will not specify, but we will tell you that it is empirically associated with A or B or C, and it must be caused by D or E or F*” (Bergner, 1997, p. 245). Then, Bergner further explained that the mentioned definition described mental disorder as a “clinically significant syndrome or pattern” without indicating what the essential characteristics of a mental disorder are, and limiting it to describe a series of elements associated with a mental disorder, such as distress, disability, and other risks, or that cause a mental disorder, such as psychological or biological dysfunction. According to the author, the DSM-IV’s (APA, 1994) definition lacks a definition of what this syndrome that is “associated with” and “caused by” effectively is. Bergner concluded that by eliminating these ambiguities, the DSM-IV’s (APA, 1994) definition could be reformulated in this way: “*mental disorder is significant personal (as opposed to situationally expectable) disability or suffering’’* (Bergner, 1997, p. 245).

One year later, Wakefield (1998) mentioned the DSM-II (APA, 1968), DSM-III (APA, 1980), DSM-IV (APA, 1994), ICD-8 (WHO, 1965), and ICD-10 (WHO, 1992). As in his previous papers (Wakefield, 1992a, 1992b), the author argued that the DSM’s definition of mental disorder presupposes that symptoms are caused by a dysfunction, and then he discussed Follette and Houts’ position on DSM’s lack of scientific progressiveness and the proposal of allowing different theoretically driven diagnostic manuals to compete (Follette & Houts, 1996), a proposal to which Wakefield replied, arguing that even if theoretical competition is essential for scientific progress, this general principle does not necessarily apply to the basic categories of a discipline. In fact, as the author explained, if each different theoretical approach defined mental disorder in its own way, the research results would not be comparable, since they may be referring to totally different conceptions of mental disorder (Wakefield, 1998). The DSM-II (APA, 1968), as an example, was not theoretically neutral, and defined disorders and the underlying processes in psychodynamic terms, making it impossible for opposing theories to empirically disconfirm the psychodynamic account of disorder. Furthermore, Wakefield argued that allowing different theoretically laden diagnostic manuals to compete would result in a regression to the so called “therapy wars” (Wakefield, 1998).

The following year, Wakefield (1999a) mentioned the DSM and replied to Follette and Houts’ (1996) critique of the DSM, according to whom the DSM is scientifically unprogressive, and different theoretical perspectives should be allowed to freely compete in defining disorders. Wakefield argued that even if competition is indeed fundamental for scientific progress, this principle does not necessarily apply to the basic categories of a discipline (such as the definition of mental disorder). Then the author argued that “*based on the overall evidence available at this time, if there is any* “*theory*” *of etiology and treatment in which it is rational to believe, it is some version of integrationism*” (Wakefield, 1999a, p. 994).

In a second paper of the same year, Wakefield (1999b) mentioned the DSM-III (APA, 1980), DSM-III-R (APA, 1987), and DSM-IV (APA, 1994). Replying to Follette and Houts’ (1996) critique of the DSM, in which they argued that both concepts of “mental disorder” and “function” are value concepts that cannot provide a scientific base for DSM nosology, Wakefield discussed his harmful dysfunction analysis, and argued that the concepts of “*mental disorder and function have scientific content that allows one to distinguish disorder from nondisorder*” (Wakefield, 1999b, p. 1001). In proposing their critique, Follette and Houts (1996) referred to a specific analysis of the concept of function proposed by Moore (1978), according to which arguments about dysfunction are based on the assumption that an organism is naturally inclined toward some end state. In this regard, Wakefield replied that judgments about what the function of a certain organism or system is, are not made on the base of some assumed end state, but rather on the evolutionary account that the function of a certain organism or system is the one by virtue of which the said organism or system has been selected by natural selection. Furthermore, both Follette and Houts (1996) and the authors of the DSM-IV (APA, 1994) highlighted that the analysis of the concept of mental disorder presented in the said diagnostic manual is aimed at evaluating “hard cases” and precisely demarcating the boundary between disorder and non-disorder, to which Wakefield replied that they are both wrong and argued that “*analyses of concepts rarely resolve boundary disputes because concepts generally are just not that precise*” (Wakefield, 1999b, p. 1012). Lastly, Wakefield strongly criticized Follette and Houts’ (1996) proposal of a behaviorist diagnostic manual as an alternative to the DSM (Wakefield, 1999b).

In a second paper of the same year, Houts (2001b) replied to Wakefield (Wakefield, 1999b, 1999c), criticized his “harmful dysfunction analysis” (Wakefield, 1992a, 1992b), and discussed how both Wakefield and the DSM-III’s (APA, 1980) first draft placed mental disorder in the domain of medical disorder. The idea of mental disorders being a subset of medical disorders first appeared in the works of Robert Spitzer, head of the DSM-III Task Force of the American Psychiatric Association, in a proposed definition of mental disorder (Spitzer & Endicott, 1978). The inclusion of such a definition in the first draft of the DSM-III (APA, 1980) caused a series of controversies between the American Psychiatric Association and the American Psychological Association, which was concerned about the jurisdiction problem raised by considering mental disorders as a subset of medical disorders. In fact, if mental disorders are medical disorders, does that mean that only physicians should be allowed to treat and study them? In response to the American Psychiatric Association’s determination to include explicit reference to the idea of mental disorders being a subset of medical disorders, the American Psychological Association established the Morley committee with the purpose of devising and disseminating an alternative manual to the DSM-III (APA, 1980). Furthermore, the American Psychological Association requested the American Psychiatric Association to include a disclaimer in the DSM-III (APA, 1980) about the scope of practice in the mental health professions. The following year, in May 1978, Robert Spitzer replied to the American Psychological Association informing them that the Task Force on Nomenclature and Statistics decided to delete any reference to mental disorders being medical disorders from the DSM-III (APA, 1980). By 1979, the American Psychological Association canceled the project of structuring an alternative manual and withdrew the request of including the cited disclaimer in the DSM-III (APA, 1980) (Houts, 2001b).

In that year, Bolton (2001) referred to the DSM-IV’s (APA, 1994) definition of mental disorder. He discussed the DSM and ICD’s choice to use the term “disorder” rather than alternatives such as “illness” or “disease”, highlighting how both the cited manuals acknowledged the issues with using such a term and making cautionary statements about this choice. Then, Bolton argued that even if the DSM-IV’s (APA, 1994) definition of mental disorder could be helpful, it contains several unexplained concepts that would require a proper definition themselves, such as “clinically significant syndrome” and “dysfunction in the individual” (Bolton, 2001). Lastly, the author argued that the “dysfunction in the individual” requirement in the DSM’s definition led to not taking into account the person/environment interaction (Bolton, 2001).

In 2005, Jablensky discussed two main critical points about the DSM-IV’s (APA, 1994) clinical significance criterion. Firstly, Jablensky highlighted that “clinical significance” understood as “desire for treatment” seems to vary considerably depending on the specific disorder considered. Secondly, he argued that the reliability of “clinical significance” assessment is generally low or modest, resulting in both false negatives and false positives. In this regard, the author proposed to separately assess disability from diagnosis in the DSM as a solution to these critical issues, similarly to what the ICD-10 (WHO, 1992) already does. Then, Jablensky discussed various issues related to the concept of comorbidity and highlighted the way in which the DSM’s categorical approach promotes artificial comorbidities. He also reported Lilienfeld et al.’s (1994) proposal to avoid the term comorbidity and embrace terms such as “diagnostic co-occurrence” and “diagnostic covariation” instead. Lastly, the author highlighted that although some disorders, such as personality disorders, seem to be better understood through a dimensional model, some other disorders, such as eating disorders, may be better understood through a hybrid one, as they are not adequately represented by either a fully categorical model or by a fully dimensional one (Jablensky, 2005).

In 2007, Horwitz mentioned the DSM-I (APA, 1952), DSM-IV (APA, 1994), and DSM-IV-TR (APA, 2000), and discussed the problem of distinguishing between distress related to mental disorder and distress that arises in “non-disordered” individuals as a response to stressful life events and environments. Then, the author highlighted that even if stressful environments can cause mental disorders (such in the case of war veterans), the DSM definition limits mental disorders to those conditions that entail disproportionate and unexpectable responses to external stressful situations. As the DSM-IV-TR (APA, 2000) states, mental disorder “*must not be merely an expectable and culturally sanctioned response to a particular event, for example the death of a loved one*” (APA, 2000, p. xxxi). Horwitz also mentioned the DSM-IV-TR’s (APA, 2000) “dysfunction in the individual” requirement and argued that the critical distinction between distress and disorder in the DSM’s definition lies in whether an internal dysfunction is responsible for the maintenance of the condition. Taking bereavement as an example, in the typical case, no internal psychological dysfunction exists, and distress in such circumstances can be considered a merely expectable response to a stressful environment. In this regard, Horwitz argued that the DSM-I (APA, 1952), by defining conditions such as a “psychoneurotic depressive reaction” as a reaction “*precipitated by a current situation, frequently by some loss sustained by the patient …*” (APA, 1952, p. 33), made it impossible to effectively separate expectable distress related to stressful environments and life events from disorder-related distress (Horwitz, 2017).

In the same year, First and Frances (2008) mentioned the DSM-III-R (APA, 1987), DSM-IV (APA, 1994), and DSM-IV-TR (APA, 2000), and presented an overview of the way the DSM’s diagnostic criteria for paraphilias changed over time. The authors criticized the DSM-IV (APA, 1994) for including the following criterion: “*the disturbance causes clinically significant distress or impairment in social, occupational, or other important areas of functioning*”, in most diagnoses, making the usual diagnostic criteria alone insufficient for identifying mental disorder. In fact, the mentioned DSM-IV (APA, 1994) criterion replaced the previous DSM-III-R (APA, 1987) criterion B for paraphilias, and criterion A also changed by adding the words “behavior” along with “fantasies” and “urges”, highlighting that behavior is often the factor that brings individuals to clinical attention. According to First and Frances, these changes resulted in two problematic consequences: first, conservative religious groups became worried that such a change meant that the DSM-IV (APA, 1994) did not recognize pedophilia as a mental disorder unless it caused distress in the individual; second, the addition of “behaviors” in criterion A led some forensic evaluators to believe that sexual offenders could be regarded as having a mental disorder solely based on having committed sexual offenses (causing a series of legal issues). Lastly, the authors argued that defining paraphilia based on behaviors makes the boundary between mental disorder and ordinary criminal acts unclear (First & Frances, 2008).

In that year, Pierre et al. (2012) discussed the challenges surrounding the DSM’s definition of mental disorder and argued that even if the cited manual provides diagnostic criteria for the mental disorders included in its classification, there still is uncertainty about the way the DSM defines mental disorder itself. Then, Pierre highlighted the DSM-IV’s (APA, 1994) statement that “*there is no assumption that each category of mental disorder is a completely discrete entity with absolute boundaries dividing it from other mental disorders or from no mental disorder*” (APA, 1994, p. xxii). Finally, even if the author praised the DSM-5′s (APA, 2013) shift toward a more dimensional approach, he argued for an even more radical dimensional shift in the DSM: “*a truly continuous view of mental health would rely less on categorical disorders and instead recognize cross-cutting symptom dimensions, not only across disorders but also along the normal–pathological continuum*” (Pierre et al., 2012, p. 656).

The following year, Schramme (2013) highlighted the DSM’s choice to use the term “disorders” rather than “diseases”, and argued that such a choice was made to avoid a definition of mental disorder that could have been too close to “somatic medicine” and would thus have required a “hard validation” of diagnostic categories that we are not currently able to provide. Schramme defended the use of the term “mental disorder” and argued that the psychological level of explanation cannot be reduced to a purely neurophysiological one. Then, the author discussed that the DSM-IV’s (APA, 1994) authors mistakenly talked out against the use of the term “mental disorders” by arguing that “mental” implies an outdated mind–body dichotomy, and highlighted that such a mistake seemed to be preserved in the DSM-5 (APA, 2013). The author concluded by stating that “*the concept of mental illness is autonomous from somatic medicine*” (Schramme, 2013, p. 8).

In 2014, Wakefield mentioned the DSM-III (APA, 1980), DSM-IV (APA, 1994), and DSM-5 (APA, 2013). In the context of confronting his harmful dysfunction analysis (Wakefield, 1992a, 1992b) with Boorse’s biostatistical theory (Boorse, 1977, 1987, 1997), the author argued that “*in psychiatry, when it is noticed that some presentation listed in the DSM is harmless, generally it is reclassified as a nondisorder*” (Wakefield, 2014, p. 674). Wakefield also highlighted that the clinical significance criterion was in fact included in the DSM to ensure that each diagnosis would be associated with a certain amount of harm, and with the purpose of eliminating harmless conditions (Wakefield, 2014).

In the same year, Jacobs (2014) mentioned the DSM-III (APA, 1980) and expressed strong criticism towards the DSM-5’s (APA, 2013) definition of mental disorder, describing it as “*vacuous, circular, and silly*” (Jacobs, 2014, p. 153).

The following year, Singh and Sinnott-Armstrong (2015) mentioned the DSM-IV-TR (APA, 2000) and specifically referred to the DSM-5’s (APA, 2013) definition of mental disorder, in which the latter is described as a “*clinically significant disturbance in an individual’s cognition, emotion regulation, or behavior that reflects a dysfunction in the psychological, biological, or developmental processes*” (APA, 2013, p. 20). In this regard, Singh and Sinnott-Armstrong examined specific diagnoses (e.g., major depressive disorder) with the purpose of analyzing the way they meet the DSM-5’s (APA, 2013) definition of mental disorder. Then, the authors expressed concern that the DSM-5’s (APA, 2013) definition may reify diagnosis due to its attributed official status, leading to misinterpretations, especially among mental health professionals who may not be fully aware of the debates surrounding such a definition. Lastly, they argued that not a single definition of mental disorder can fully satisfy all the purposes, whether clinical, ethical, or legal (Singh & Sinnott-Armstrong, 2015).

In that period, Troisi (2015) mentioned the DSM-IV (APA, 1994) and specifically referred to the DSM-5’s (APA, 2013) definition of mental disorder. The author argued that such a definition is based on the following criteria for morbidity: dysfunction, distress, and disability. Then, Troisi discussed each of these criteria by summarizing how they have been conceptualized by the DSM and other authors, and analyzed their validity from the perspective of Darwinian psychiatry. Troisi strongly criticized the DSM-5’s (APA, 2013) definition of mental disorder and argued that even if the cited definition is based on the concept of dysfunction, by failing to define what a dysfunction effectively is, the DSM provides a definition that is “*not only vague but also tautological: the presence of a mental disorder reflects an underlying dysfunction, and the presence of a dysfunction defines a mental disorder*” (Troisi, 2015, p. 325). Troisi also argued that the DSM-5 (APA, 2013) implies a “*Cartesian view of the mind–body problem, assuming that psychological and biological processes are separable and entirely distinct realms*” (Troisi, 2015, p. 325) and a medical model, thus contradicting its declared atheoreticality (Troisi, 2015).

During the same time, Bergner and Bunford referred to the DSM-5’s (APA, 2013) definition of mental disorder and discussed the way in which the concept of dysfunction is portrayed. In this regard, the authors highlighted that in the DSM-5 (APA, 2013), a disorder is not a dysfunction, but is rather defined as “*a syndrome characterized by clinically significant disturbance in an individual’s cognition, emotion regulation, or behavior*” (APA, 2013, p. 20), and that this syndrome “*reflects a dysfunction in the psychological, biological, or developmental processes underlying mental functioning*” (APA, 2013, p. 20). Bergner and Bunford thus argued that “disorder” and “dysfunction” are described as two different entities, between which a vague causal relation is implied. Then, they argued that by rigorously applying the DSM-5’s (APA, 2013) definition of mental disorder, one would not be able to make a diagnosis until the clinician had established the presence of a syndrome characterized by “clinically significant disturbance”, and that such a syndrome “reflects” a dysfunction “in psychological, biological, or developmental processes” (Bergner & Bunford, 2017). Thus, it would not be theoretically possible to diagnose a person with a condition even when the listed criteria for a disorder are met, unless a syndrome of disturbance reflecting a dysfunction were established to be present, with the result of making it extremely difficult, if not impossible, to provide a diagnosis (Bergner & Bunford, 2017). Lastly, the authors argued that the DSM-5’s (APA, 2013) definition of mental disorder is not an actual definition, since a proper definition, in the strict sense of the term, requires a careful specification of the necessary and sufficient conditions for the correct designation of a term (Bergner & Bunford, 2017).

The following year, Telles-Correia et al. (2018) mentioned the DSM-II (APA, 1968) and referred to the DSM-III (APA, 1980) and DSM-5’s (APA, 2013) definitions of mental disorder. The authors argued that the DSM-III’s (APA, 1980) definition was the first formal definition of mental disorder, and highlighted that the definition was specifically designed to address various issues such as the need for an atheoretical and evidence-based classification of mental disorders, the removal of homosexuality from the classification, and the necessity of countering antipsychiatry arguments. Telles-Correia et al. argued that the DSM-III’s (APA, 1980) definition privileged the harm criteria, encompassing distress and disability, while avoiding terms such as “dysfunction”. As stated by Spitzer and Endicott, “*these criteria avoid such terms as* “*dysfunction*,” “*maladaptive*,” *or* “*abnormal*”, *terms which themselves beg definition*” (Spitzer & Endicott, 1978, p. 17). The harm criteria remained fundamental in the DSM-IV’s (APA, 1994) definition too, but in the DSM-5 (APA, 2013), the concept of dysfunction took precedence in defining mental disorder, so that distress and disability were regarded as frequent characteristics of disorder but not necessary conditions. The author argued that such change could expose psychiatry to “*the danger that entities considered psychological or biological dysfunctions, according to certain theoretical currents (easily permeable to moral and social values), may be considered mental disorders*” (Telles-Correia et al., 2018, p. 3).

In 2019, Amoretti and Lalumera mentioned the DSM-III-R (APA, 1987), DSM-IV (APA, 1994), and DSM-IV-TR (APA, 2000), and specifically referred to the DSM-5’s (APA, 2013) definition of mental disorder. The authors critically examined the DSM-5’s (APA, 2013) definition and highlighted two key requirements: dysfunction and harm (in terms of distress or disability). Amoretti and Lalumera discussed how the DSM-5’s (APA, 2013) definition makes dysfunction a necessary criterion for mental disorder, also making the further step of regarding it as the pathological cause and implying that in the absence of an underlying dysfunction, a condition cannot be identified as a mental disorder (Amoretti & Lalumera, 2019). With regard to the harm criterion, encompassing distress and disability, those are regarded by the DSM-5 (APA, 2013) as frequent characteristics of mental disorder, but not necessary conditions, suggesting that a mental disorder can be correctly diagnosed even if not harmful. The authors also distinguished between the so-called “mental disorder tokens” and “mental disorder types”, and argued that while the harm requirement may not be necessary for the general DSM’s definition of mental disorder, it could be more useful as a specific diagnostic criterion for certain individual mental disorders. Amoretti and Lalumera concluded their discussion by indicating that the harm requirement should not be included in the DSM-5’s (APA, 2013) general definition of disorder (Amoretti & Lalumera, 2019).

In 2021, Stein et al. mentioned the DSM-III (APA, 1980), DSM-IV (APA, 1994), DSM-5 (APA, 2013), ICD-10 (WHO, 1992), and ICD-11 (WHO, 2022). The authors discussed the DSM’s definitions of mental disorder and proposed an analysis of three different conceptual cases: (1) entities associated with harm, but for which there is limited evidence of underlying dysfunction, (2) entities involving dysfunction but without strong evidence that they produce harm, and (3) entities involving possible harm and dysfunction, and thus possibly indicative of a disorder, but which are controversial for various reasons. Stein et al. concluded that “*decisions about the introduction of new entities into the nosology need to balance the harm to the individual with harm to society*” (Stein et al., 2021, p. 899) and that “*there may be significant debate about the extent to which harm is due to the failure of society to accommodate differences*” (Stein et al., 2021, p. 899). They also proposed various changes for the DSM-5’s definition of mental disorder, such as referring to “*psychobiological dysfunction*” instead of “*psychological, biological, or developmental processes*”, aiming to emphasize that psychology and biology are intertwined (Stein et al., 2021). Second, they proposed that “*the consequences of a mental disorder are clinically significant distress … or disability …*” (Stein et al., 2021, p. 895). The authors also proposed that mental disorders must have “*diagnostic validity on the basis of various diagnostic validators (e.g., prognostic significance, psychobiological disruption, response to treatment)*” (Stein et al., 2021, p. 895). For a complete discussion of all the proposed changes, see (Stein et al., 2021).

The following year, Biturajac and Jurjako (2022) mentioned the DSM-III (APA, 1980), DSM-IV (APA, 1994), and ICD-10 (WHO, 1992), and referred to the DSM-5’s (APA, 2013) definition of mental disorder. The authors discussed the demotion of the harm component in the DSM’s definition of mental disorder and highlighted that while in the DSM-IV (APA, 1994), the definition of harm was a necessary feature of mental disorders, in the DSM-5 (APA, 2013), harm is described as something that is “usually associated” with but not necessary for mental disorders (Biturajac & Jurjako, 2022). Then, Biturajac and Jurjako argued that such a shift represents a move towards a dysfunction-only conceptualization of mental disorders, allowing mental disorders to be identified solely based on the presence of a dysfunction, even when the latter is not causing any harm (Biturajac & Jurjako, 2022). They also highlighted a series of advantages in maintaining the harm component in the DSM’s definition (Biturajac & Jurjako, 2022). According to the authors, requiring that a condition must not only involve dysfunction but also harm, would avoid individuals being regarded as having a disorder simply for deviating from social norms or exhibiting socially deviant behavior. Furthermore, Biturajac and Jurjako argued that retaining the notion of harm in the definition helps mental health professionals to better distinguish between pathological and nonpathological conditions (Biturajac & Jurjako, 2022).

In summary, such a debate on the DSM’s proposed definitions proceeded in parallel with the more general debate on the definition of mental disorder, which started with the first formal definition proposed by Spitzer and Endicott (1978). Among the papers that participated the debate, some papers analyzed in detail different points about the DSM’s definition, such as the concept of “clinically significant” (Wakefield, 1992a, 1992b, 2014; Bolton, 2001; Jablensky, 2005), the concepts of “harm”, “distress”, and “disability” (Wakefield, 1992a, 1992b; Follette & Houts, 1996; Houts, 2001a, 2001b; Bolton, 2001; Telles-Correia et al., 2018; Horwitz & Wakefield, 2007), and the so-called “dysfunction in the individual” requirement (Wakefield, 1992a, 1992b). Moreover, another frequently discussed point is to do with the problem of distinguishing between “socially deviant behavior” and “mental disorder” (Troisi, 2015; Frances et al., 2008).

## 5. Some Critics on the Six Definitions of MD Proposed in the DSM

The DSM’s definitions also attracted some minor and major critiques, like the one proposed by Troisi (2015), who labeled the DSM’s definitions as “*vague and tautological*” and highlighted the problematic tendency to identify MD based on the presence of dysfunction, while at the same time inferring the presence of dysfunction based on the presence of MD (“*the presence of a mental disorder reflects an underlying dysfunction, and the presence of a dysfunction defines a mental disorder*” (Troisi, 2015, p. 325).

Then, some other critical issues, like the problem of distinguishing between “disorder” and “responses to life events”, which had been addressed by Crowe (“authoritative image of normality pervades many areas of social life and pathologises experiences that could be regarded as responses to life events”) (Crowe, 2000, p. 69).

Another discussed issue was concerned with the importance of balancing between the “*flexibility*” and “*specificity*” of the MD definition in order to satisfy the needs of the diverse DSM users, which include “*clinicians in diagnosis and management, but also by many other people, including patients, families, researchers…*” (see Singh & Sinnott-Armstrong, 2015 for a discussion on this topic).

Another addressed issue was concerned with the need to prevent the “*risk of reification of mental disorder*” (see Singh & Sinnott-Armstrong, 2015 for a discussion on this theme).

Moreover, the concept of “*underlying dysfunction*” remains a very critical theme in almost all the known mental disorders, with few exceptions, as well as the “*risk of reification*” and the need to balance between the “*flexibility*” and “*specificity*” of the MD definition. These, along with other critical themes, remain central to the debate (Hyman, 2010).

## 6. Conclusions and Clinical Implications

In this perspective paper, we focused on the definitions of “mental disorder” proposed in the various DSM editions, starting from the third edition of 1980. According to our analysis, over about 42 years, the various American Psychiatric Association’s working groups, responsible for the development of the DSM, produced six definitions of mental disorder that shared a similar structure and approach, with only slight changes. We discussed the general matrix/structure of the proposed definitions, as well as the main similarities and/or divergences, and some of the specific constructs and approaches used in such definitions. Additionally, we examined some papers that addressed the same topic in a similar manner and discussed the general debate that accompanied the proposal of the various DSM definitions, the significant attention those definitions attracted, as well as some minor and major criticisms.

Regarding the clinical implications of this paper, bearing in mind all those issues, we believe that there is still a lot of work to be carried out on the definition of mental disorder itself, but also on the practical effects of any definition. We hope that the insights gained from previous and current analysis and debates might pave the way for future research to address the current limitations and advance the understanding of the construct “mental disorder” according to the perspective of clinical psychology…

In our view, further research is needed in two main different but interrelated directions: a general framework, useful for understanding both continuity and discontinuity among the psychopathological features during the entire lifecycle, also taking into account individual, relational, social, and environmental protective and risk factors; and an overall approach, useful for intervention, and able to take into account all the previously discussed aspects (Petretto et al., 2025). A lifespan approach in psychopathology (Cicchetti & Sroufe, 2000; Cicchetti, 2016; Venta et al., 2021), like the one related to developmental psychopathology, that is focused on all the phases of the lifecycle, rather than on the specific critical steps of an individual’s life, might offer a new way for thinking about causation, within a complex multilevel causal model, characterized by interdependence among the different levels and by mutually reinforcing effects, operating over time. We also need a new way to think about prevention and intervention. Last, but not least, the described approach could offer a new way of conceptualizing specific concepts, if needed (like in the case of the concept of mental disorder itself).

## Figures and Tables

**Table 1:** Definitions of mental disorders in DSM.

**DSM-III** **(APA, 1980)**	“In DSM-III each of the mental disorders is conceptualized as a clinically significant behavioral or psychological syndrome or pattern that occurs in an individual and that is *typically associated with either a painful symptom (distress) or impairment in one or more important areas of functioning (disability)*. In addition, *there is an inference* that there is a behavioral, psychological, or biological dysfunction, and that the disturbance is not only in the relationship between the individual and society. (When the disturbance is limited to a conflict between an individual and society, this may represent social deviance, which may or may not be commendable, but is not by itself a mental disorder.)”
**DSM-III-R** **(APA, 1987)**	“In DSM-III-R each of the mental disorders is conceptualized as a clinically significant behavioral or psychological syndrome or pattern that occurs in a person and that is *associated with present distress (a painful symptom) or disability (impairment in one or more important areas of functioning) or with a significantly increased risk of suffering death, pain, disability, or an important loss of freedom*. In addition, this syndrome or pattern must not be merely an expectable response to a particular event, e.g., the death of a loved one. *Whatever its original cause*, it must currently be considered a manifestation of a behavioral, psychological, or biological dysfunction in the person. Neither deviant behavior, e.g., political, religious, or sexual, nor conflicts that are primarily between the individual and society are mental disorders unless the deviance or conflict is a symptom of a dysfunction in the person, as described above.”
**DSM-IV** **(APA, 1994)**	“In DSM-IV, each of the mental disorders is conceptualized as a clinically significant behavioral or psychological syndrome or pattern that occurs in an individual and that is *associated with present distress (e.g., a painful symptom) or disability (i.e., impairment in one or more important areas of functioning) or with a significantly* increased risk of suffering death, pain, disability, or an important loss of freedom. In addition, this syndrome or pattern must not be merely an expectable and culturally sanctioned response to a particular event, for example, the death of a loved one. *Whatever its original cause*, it must currently be considered a manifestation of a behavioral, psychological, or biological dysfunction in the individual. Neither deviant behavior (e.g., political, religious, or sexual) nor conflicts that are primarily between the individual and society are mental disorders unless the deviance or conflict is a symptom of a dysfunction in the individual, as described above.”
**DSM-IV-TR** **(APA, 2000)**	“In DSM-IV, each of the mental disorders is conceptualized as a clinically significant behavioral or psychological syndrome or pattern that *occurs in an individual and that is associated with present distress (e.g., a painful symptom) or disability (i.e., impairment in one or more important areas of functioning) or with a significantly increased risk of suffering death, pain, disability, or an important loss of freedom*. In addition, this syndrome or pattern must not be merely an expectable and culturally sanctioned response to a particular event, for example, the death of a loved one. *Whatever its original cause*, it must currently be considered a manifestation of a behavioral, psychological, or biological dysfunction in the individual. Neither deviant behavior (e.g., political, religious, or sexual) nor conflicts that are primarily between the individual and society are mental disorders unless the deviance or conflict is a symptom of a dysfunction in the individual, as described above.”
**DSM-5** **(APA, 2013)**	“A mental disorder is a syndrome characterized by clinically significant disturbance in an individual’s cognition, emotion regulation, or behavior that reflects a dysfunction in the psychological, biological, or developmental processes underlying mental functioning. Mental disorders are usually associated with significant distress or disability in social, occupational, or other important activities. An expectable or culturally approved response to a common stressor or loss, such as the death of a loved one, is not a mental disorder. Socially deviant behavior (e.g., political, religious, or sexual) and conflicts that are primarily between the individual and society are not mental disorders unless the deviance or conflict results from a dysfunction in the individual, as described above.”
**DSM-5-TR** **(APA, 2022)**	“A mental disorder is a syndrome characterized by clinically significant disturbance in an individual’s cognition, emotion regulation, or behavior that reflects a dysfunction in the psychological, biological, or developmental processes underlying mental functioning. Mental disorders are usually associated with significant distress or disability in social, occupational, or other important activities. An expectable or culturally approved response to a common stressor or loss, such as the death of a loved one, is not a mental disorder. Socially deviant behavior (e.g., political, religious, or sexual) and conflicts that are primarily between the individual and society are not mental disorders unless the deviance or conflict results from a dysfunction in the individual, as described above.”

**Table 2:** Similarities and differences in the definitions of mental disorders in DSMs.

**Similarities Between the DMSs’ Definitions of MD**
Similar overall structure of the definition; Overall description of MD’s features; Overall description of consequences of MD in individual life; Overall description underlying mechanisms; Distinction between MD and other kinds of human facts/behaviors and conditions.
**Differences Between the DMSs’ Definitions of MD**
Terminologies and semantics used in each definition changed (regarding the levels of psychological functioning and domains, the mechanisms/processes underlying mental disorders, and the overall and specific consequences of MD on individuals’ lives); Some words appeared, then disappeared and later made a comeback, and a few words seem to be used in such an interchangeable manner (like in the cases of the words “pain” and “distress” or “impairment” and “disability”); The same idea seems to have been expressed with different adverbs (e.g., the use of “usually” or “typically” to describe the relationship between MD’s features and the consequences of MD on individuals’ lives).

## Abbreviation

The following abbreviation is used in this manuscript:

MD	Mental disorder(s)
